# Evaluation of Publication Growth in Pediatric Dentistry From 2005 to 2024: A Bibliometric Analysis

**DOI:** 10.7759/cureus.100546

**Published:** 2026-01-01

**Authors:** Pillai Arun Gopinathan, Ikram UI Haq, Bijesh Yadav, Atheer Haif Alotaibi, Abdulaziz Abdulmohsen Alqahtani, Sultan Abdullah Alotaibi, Saud Shaher Almutairy, Ibrahim Dkheel Alanazi

**Affiliations:** 1 Maxillofacial Surgery and Diagnostic Sciences, College of Dentistry, King Saud Bin Abdulaziz University for Health Sciences, Ministry of National Guard Health Affairs, King Abdullah International Medical Research Center (KAIMRC), Riyadh, SAU; 2 Dentistry, College of Dentistry, King Saud Bin Abdulaziz University for Health Sciences, Riyadh, SAU; 3 Population Health, Division of Biostatistics, King Abdullah International Medical Research Center (KAIMRC), Riyadh, SAU

**Keywords:** bibliometric, citations, pediatric dentistry, research productivity, vosviewer

## Abstract

The study aimed to evaluate global research output, impact, collaboration, and thematic trends in pediatric dentistry (PD) from 2005 to 2024, utilizing data from the Web of Science (WoS). A bibliometric investigation was conducted on 4,032 articles and reviews indexed in the WoS Core Collection. The indicators assessed included publication growth, citation patterns, authorship and institutional productivity, contributions by country, journal performance, and keyword co-occurrence. The R Foundation and VOSviewer software were utilized for data analysis. In the results, research output increased steadily from 35 papers in 2005 to 508 in 2024, reflecting an average annual growth rate of 16.87%. The most productive countries were the United States, Brazil, and India, while Australia, England, and Germany achieved the highest citation impact. Specialized dental journals accounted for the majority of the output and citations. Collaborative authorship was prevalent, with mid- to large-sized teams producing the most influential research. A keyword analysis indicated that core themes included dental caries, preventive dentistry, clinical management, and child-focused oral health. A significant relationship was observed between accessibility modes, document types, and journal types (p < 0.001). Despite this strong growth, contributions were uneven across different countries and institutions, and many authors published only a single paper. In conclusion, PD research has significantly expanded over the past two decades, becoming more collaborative and globally diverse. However, disparities in productivity and impact continue to exist across regions. To enhance understanding of global research trends in PD, it is recommended that future studies employ broader search strategies, utilize additional databases, and incorporate qualitative assessments.

## Introduction and background

Pediatric dentistry (PD) is a specialized field focused on addressing the oral health needs of children, from infancy through adolescence. It encompasses the prevention, diagnosis, and management of dental conditions in young patients, placing particular emphasis on behavioral guidance, growth and development, and age-appropriate care [1-3]. The discipline prioritizes preventive measures, including oral hygiene education, dietary guidance, and early interventions aimed at reducing the risk of dental caries and other oral diseases [4,5]. By combining clinical expertise with a child-centered approach, PD plays a crucial role in fostering healthy oral habits and promoting long-term oral health [6,7].

Bibliometric investigation provides a quantitative approach to trace the structure, development, and impact of scientific literature [8,9]. The application of bibliometrics to PD allows for the identification of periodic growth, highlights prominent publication channels, and reveals leading countries, institutions, authors, and research themes, as well as collaboration patterns. This information is essential for researchers, educators, funders, and policymakers seeking to prioritize future research and strengthen evidence-based clinical practice [10,11]. The earliest notable bibliometric study in PD was conducted by Nainar, who analyzed 30 years of publication records (1969-1988) from two dental journals. This study found that the majority of papers (71%) were descriptive studies or case reports (Level III evidence), while only 6% were randomized controlled trials [12]. Yang et al. identified 8,097 PD records indexed in PubMed between 1989 and 1998; however, only 15.72% (n = 1,237) specifically focused on children under 12 years of age [13]. Garcovich et al. examined the 100 most cited PD articles published between 1967 and 2013, with nearly three-quarters appearing in two journals: *Pediatric Dentistry* (n = 45) and the *International Journal of Paediatric Dentistry* (n = 27) [14]. Poletto et al. analyzed 572 papers published in the *Brazilian Journal of Pediatric Dentistry* from 1998 to 2007, finding that case reports (33%), cross-sectional studies (30%), and literature reviews (23%) were the predominant study designs. Most of the papers originated from São Paulo State (40%), followed by Rio de Janeiro State (17%) [10]. Ohta et al. (2020) assessed 1,311 papers published in the *Pediatric Dentistry* journal between 1999 and 2018, identifying cariology (12.7%) as the leading research area, with the United States as the most productive contributor [11]. García et al. reviewed 3,027 papers published from 2008 to 2020 in four PD journals indexed in the 2020 Journal Citation Reports [15]. Zhang et al. examined 396 papers on pediatric dental sedation published from 1993 to 2022, highlighting that the United States contributed the largest share at 35.4%. The most frequent publication venue was *Pediatric Dentistry*, with 85 articles. The Federal University of Goiás produced the highest number of papers, while the University of Washington achieved the greatest citation impact [16]. Mahyaddinova et al. analyzed 342 papers on neurodevelopmental disorders in PD, noting that one-third of the research originated from the United States. Most of these studies concentrated on oral health assessments, epidemiological patterns, and behavior-oriented treatment approaches [17]. Gülşen et al. identified 78 publications concerning artificial intelligence applications in PD, with the United States leading with 17 papers, followed by Turkiye and China [18]. Tasdemir reviewed 1,949 PD papers produced in Turkiye from 1984 to 2024, reporting that the *Journal of Clinical Pediatric Dentistry* published the highest number. Hacettepe University was recognized as the most productive institution, while international collaboration appeared in only 5% of publications, primarily with the United States [19]. Similarly, Bakhsh et al. evaluated 2,574 papers published in five Saudi dental journals from 2009 to 2023, finding that only 49 (5.74%) addressed pediatric dentistry [20].

The primary objectives of this study were to describe trends in publication and citation over time; to assess the modes of accessibility, types of documents, and characteristics of journals; to identify the leading journals, most productive countries, institutions, and authors, along with patterns of authorship and average authorship over time; to highlight prevalent languages and major funding agencies; and to uncover the most frequently occurring thematic topics within PD. The findings aim to inform researchers about collaboration opportunities, guide educators and program directors in aligning curricula with evolving evidence, and assist funders and policymakers in targeting investments toward areas of high impact and unmet need.

The literature review highlights the necessity for a comprehensive global bibliometric evaluation of PD. By concentrating on articles and reviews indexed in the widely used Web of Science (WoS) database, this study offers a reproducible and consistent representation of global research activity and influence in PD from 2005 to 2024. This research seeks to fill that gap by systematically analyzing trends, productivity, and significant emerging themes.

## Review

Methodology

The study utilized a dataset obtained from the Core Collection, a key component of the WoS, an online bibliographic and citation indexing database maintained by Clarivate Analytics. Data retrieval was conducted in November 2025. For the advanced search in WoS, the “Topics” (TS) field was used with the following query:

TS=("Pediatric* Dentistry") OR TS=("Paediatric* Dentistry").

**Figure 1:**
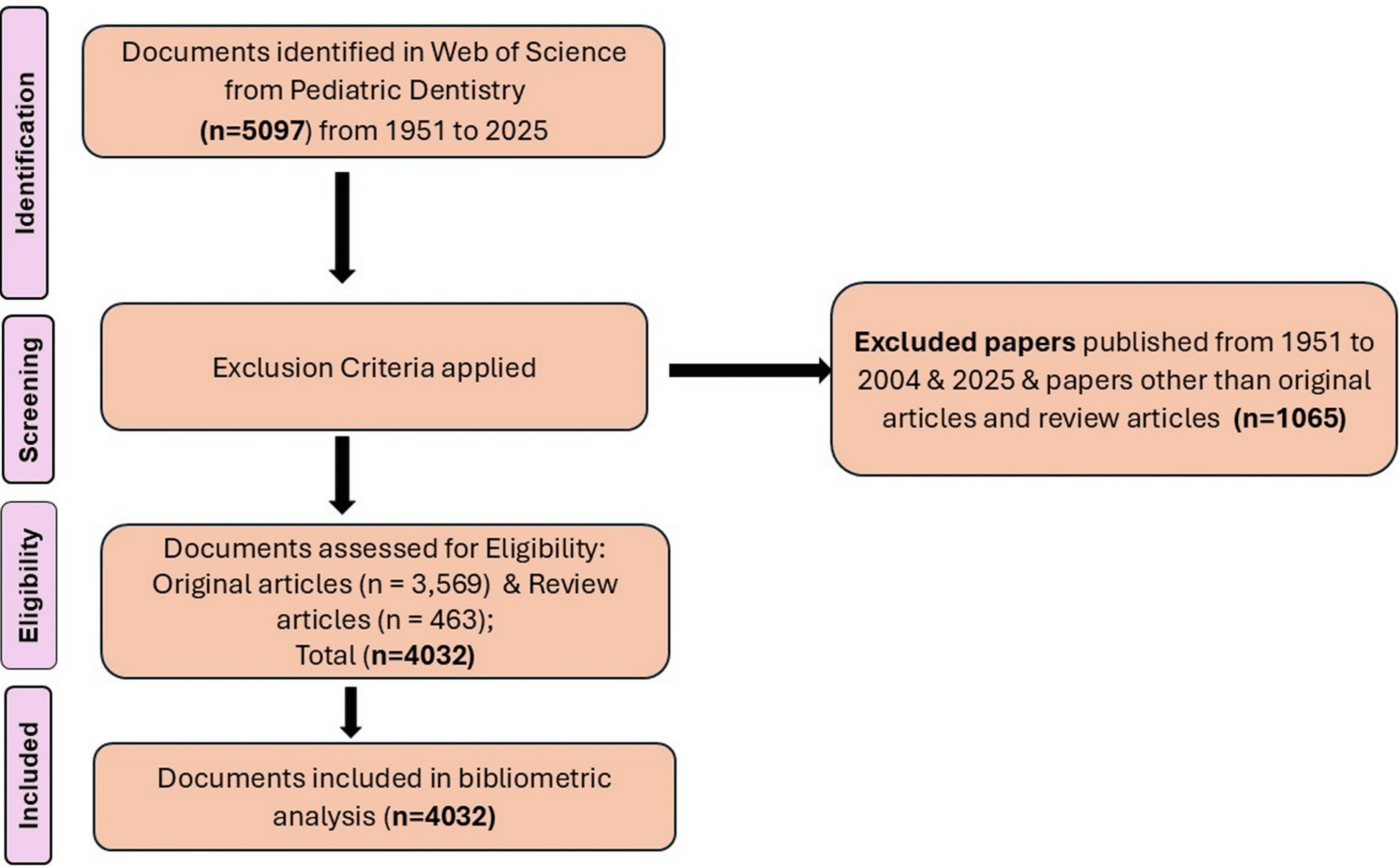
Screening process of articles

The initial search yielded 5,097 records published from 1951 to the date of data extraction (December 4, 2025). Applying the year filter, the publications were concentrated from 2005 to 2024, excluding 228 publications before 2005 due to low productivity. Additionally, 560 publications from 2025 were excluded, as that year was still ongoing, and recent publications generally receive fewer citations. The document-type filters were later applied, selecting original articles (n = 3,569) and review articles (n = 463), while excluding 277 publications of other types, which were editorial materials (n = 139), proceeding papers (n = 36), letters (n = 36), early access articles (n = 27), meeting abstracts (n = 20), corrections (n = 7), news items (n = 7), book reviews (n = 6), books (n = 2), retracted publications (n = 2), and one book chapter. In total, 1,065 publications were excluded. Consequently, 4,032 papers were selected for data analysis, which collectively garnered 46,978 citations, resulting in an average of 19.09 citations per paper. The citation impact is defined as the average citations received per article, calculated by dividing total citations by the number of articles. A flow chart for the selection criteria of articles is depicted in Figure 1.

Data, Bibliometric Software, and Statistical Analysis

The review study was conducted with VOSviewer (version 1.6.10, Centre for Science and Technology Studies, Leiden University, Netherlands) and R software, v4.4 (R Foundation for Statistical Computing, Vienna, Austria). The results were presented as a 95% confidence interval proportional difference, with a p-value of less than 0.05 considered significant. The proportion test was employed to evaluate the significance across accessibility modes, document kinds, and journal types.

Results

Publication Growth by Years

The publication trend in PD demonstrates a steady and significant increase in research output. The number of papers grew from 35 in 2005 to a peak of 508 in 2024, reflecting considerable growth in scholarly activity over the two decades, as detailed in Table 1. Initial progress was gradual, with approximately one-fourth of the papers (n = 979; 24.28%) published during the first decade. However, publication growth accelerated notably after 2014, resulting in three-fourths of the papers being published in the second decade. This trend indicates a rising interest in PD, enhanced research capabilities within dental institutions, and greater involvement from the dental research community. The overall average growth rate was recorded at 16.87%, confirming a positive long-term upward trend.

**Table 1:** Growth of papers and citations by years with average growth rate The asterisk indicates the average citation impact and average annual growth rate of pediatric dentistry publications from 2005 to 2024.

Year	Total Papers	Total Citation	Citation Impact	Annual Growth Rate
2005	35 (0.87%)	1076	30.74	
2006	45 (1.12%)	1404	31.20	28.57
2007	74 (1.84%)	1558	21.05	64.44
2008	75 (1.86%)	1567	20.89	1.35
2009	109 (2.70%)	2835	26.01	45.33
2010	93 (2.31%)	2168	23.31	-14.68
2011	96 (2.38%)	2008	20.92	3.23
2012	124 (3.08%)	2303	18.57	29.17
2013	153 (3.79%)	2831	18.50	23.39
2014	175 (4.34%)	2531	14.46	14.38
2015	144 (3.57%)	2269	15.76	-17.71
2016	177 (4.39%)	3097	17.50	22.92
2017	195 (4.84%)	2747	14.09	10.17
2018	243 (6.03%)	3241	13.34	24.62
2019	248 (6.15%)	3693	14.89	2.06
2020	346 (8.58%)	3421	9.89	39.52
2021	390 (9.67%)	3222	8.26	12.72
2022	416 (10.32%)	2430	5.84	6.67
2023	386 (9.57%)	1565	4.05	-7.21
2024	508 (12.60%)	1012	1.99	31.61
Total/Average*	4,032	46,978	19.09*	16.87*

Citation patterns indicate that the highest citation counts occurred between 2009 and 2014. Papers published in the first decade had more time to accumulate citations and likely addressed foundational or evolving themes that attracted significant attention. In contrast, papers published in the second decade experienced fluctuating citation counts, and in recent years (2021-2024), these counts have declined sharply. This decrease does not reflect weak research quality; rather, it illustrates the normal citation delay, as newer papers require time to gain visibility and citations.

The analysis of citation impact, or average citations per paper, further supports this clarification. Citation impact was highest in the earlier years, while it has steadily declined in recent years. This decline follows a typical bibliometric pattern observed when publication volume increases sharply; although recent research output is substantial, it has not yet had sufficient time to mature in terms of citation accumulation.

Table 2 examines three bibliometric indicators: accessibility mode, document types, and the nature of journals, along with the total number of papers and citation impact. The first variable shows that, out of the total papers, 43.5% (n = 1754) were published in open-access mode, and 56.5% (n = 2278) were subscription-based. Subscription-based papers make up a slightly larger share, with an average of 12.47 citations per paper, as compared to open-access papers, which gained an average of 10.59 citations per paper. There was a 1.88% (95% CI: 1.29%-2.47%) significant difference in this category of the open-access vs subscription-based journals (p < 0.001). This suggests that although open-access papers increase visibility, subscription-based papers may attract more influential attention from the research community.

**Table 2:** Distribution of papers and citations by accessibility mode, document types, and journal types *p < 0.05: Statistically significant.

Variables	Subvariables	Total Papers	Total Citations	Citation Impact	Difference (95% CI)	P-value
Accessibility mode	Open-accessed papers	1,754 (43.50%)	18,579	10.59	1.88 (1.29, 2.47)	<0.001
	Subscription-based papers	2,278 (56.50%)	28,399	12.47		
Document types	Original articles	3,569 (88.50%)	37,598	10.53	9.73 (8.86, 10.6)	<0.001
	Review articles	463 (11.50%)	9,380	20.26		
Journal types	Dental journals	2,939 (73%)	36535	12.43	2.87 (2.21, 3.53)	<0.001
	Non-dental journals	1093 (27%)	10,453	9.56		

Regarding document types, the majority of publications (88.5%) were original research articles (n = 3569), while review articles account for only 11.5% (n = 463). Despite their lower quantity, review articles demonstrated a significantly higher average of 20.25 citations per paper, compared to regular research articles with an average of 10.53 citations per paper. There was a 9.73% (95% CI: 8.86%-10.6%) significant difference in this category of original articles vs review articles (p < 0.001). This observation aligns with typical scholarly patterns, as review papers often synthesize existing knowledge, leading to greater citation rates due to their broader relevance (Table 2).

In terms of journal types, 73% (n = 2939) of papers were published in dental journals, while 27% (n = 1093) appeared in non-dental journals. Dental journals exhibit a higher average of 12.43 citations per paper as compared to the non-dental journals, which gained an average citation of 9.56 per paper, suggesting that research published in core disciplinary journals tends to be more visible and frequently cited. There was a 2.87% (95% CI: 2.21%-3.53%) significant difference in this category of dental vs non-dental journals (p < 0.001). This may result from a better alignment with the target readership and greater relevance to specialized researchers (Table 2).

Core Research Journals

The selected papers were published in 552 different sources, with over half of these sources (n = 298; 54%) contributing just one paper each. About 65 sources published 10 or more papers. Approximately 44% (n = 1,760) of the total papers appeared in these top journals. An analysis of the top 10 frequently used sources reveals notable variation in both productivity and scholarly influence within PD research. Table 3 depicts the distribution of the top 10 journals. The *Pediatric Dental Journal and Pediatric Dentistry* are the leading publishers, with 311 and 277 papers, respectively; however, their citation impacts differ significantly. *Pediatric Dentistry* claims a stronger average of 13.59 citations per paper, whereas the *Pediatric Dental Journal* has a much lower average of 3.15 citations per paper. Among all journals, the *International Journal of Paediatric Dentistry* is distinguished by having one of the highest average citation (22.21) per paper, despite publishing fewer papers than the top two journals, indicating strong visibility and influence of its research. A similar trend is evident for the *European Archives of Paediatric Dentistry*, which maintains a high average citation of 20.32 per paper. In contrast, journals like *Pesquisa Brasileira em Odontopediatria e Clinica Integrada* and the *Journal of Dentistry for Children* exhibit lower average citations (2.37 and 4.13) per paper, suggesting they have a more limited global reach. High-impact-factor journals, such as *BMC Oral Health* (IF 3.1), also show commendable average citation of (11.46) per paper, even with fewer publications. Overall, the analysis indicates that journals with a moderate number of papers often achieve the strongest scholarly influence, while some high-output journals demonstrate lower citation impacts, reflecting differences in audience, scope, and research quality across various outlets.

**Table 3:** Top 10 frequently used journals

Serial No.	Name of Journal	Impact Factor	Total Papers	Total Citations	Citation Impact
1.	Pediatric Dental Journal	0.8	311 (7.71%)	980	3.15
2.	Pediatric Dentistry	1.7	277 (6.87%)	3,764	13.59
3.	International Journal of Paediatric Dentistry	1.9	270 (6.70%)	5,998	22.21
4.	European Archives of Paediatric Dentistry	2	213 (5.28%)	4,329	20.32
5.	Journal of Clinical Pediatric Dentistry	2.2	147 (3.67%)	1,095	7.45
6.	Journal of Dentistry for Children	0.5	133 (3.30%)	549	4.13
7.	European Journal of Paediatric Dentistry	2.7	120 (2.98%)	1,293	10.78
8.	Journal of Dental Education	1.6	102 (2.53%)	1,026	10.06
9.	Pesquisa Brasileira em Odontopediatria e Clinica Integrada	0.4	98 (2.43%)	232	2.37
10.	BMC Oral Health	3.1	89 (2.21%)	1,020	11.46

Top Countries

A total of 4,032 papers on PD have been produced by authors from 103 countries. Only 12 countries contributed to a single paper, while authors from 91 countries produced more than one paper, and 58 countries recorded 10 or more papers. Details of the top 10 most productive countries are listed in Table 4. The United States contributed the highest number of papers (n = 756; 18.75%), followed by Brazil with 494 (12.25%) and India with 349 (8.65%) papers. Although Australia produced 115 (2.85%) papers, it achieved the highest average of 31.55 citations per paper, followed by England (18.82) and Germany (18.5).

**Table 4:** Top 10 productive countries

Serial No.	Country	Total Papers	Total Citations	Citation Impact
1.	United States	756 (18.75%)	11,665	15.43
2.	Brazil	494 (12.25%)	6,345	12.84
3.	India	349 (8.66%)	1,630	4.67
4.	Turkiye	292 (7.24%)	2,504	8.58
5.	England	285 (7.07%)	5,363	18.82
6.	Italy	216 (5.36%)	2,448	11.33
7.	Japan	200 (4.96%)	849	4.25
8.	Saudi Arabia	178 (4.41%)	1,543	8.67
9.	Australia	115 (2.85%)	3,628	31.55
10.	Germany	113 (2.80%)	2,091	18.5

Top Research Producing Organizations

A total of 3,200 organizations have been identified, and two-thirds (n = 2,121; 66.28%) of these organizations contributed to a single paper. Additionally, 156 organizations produced 10 or more papers. The top 10 productive organizations are listed in Table 5. Universidade de São Paulo ranked first with 118 papers, followed by the University System of Ohio with 98 papers and the University of London with 96 papers. The University of Washington had the highest research impact, with its papers receiving an average of 26.04 citations per paper, followed by the University of North Carolina (24.21) and the University of London (18.7). The Saveetha Institute of Medical Technical Science in India received the least attention, with an average citation of 4.0 per paper.

**Table 5:** Top 10 productive organizations

Serial No.	Name of Organization	Total Papers	Total Citations	Citation Impact
1	Universidade de São Paulo, Brazil	118 (2.93%)	1,384	11.73
2.	University System of Ohio, United States	98 (2.43%)	1,656	16.9
3.	University of London, England	96 (2.38%)	1,795	18.7
4.	University of Washington, United States	78 (1.93%)	2,031	26.04
5.	University of North Carolina, United States	68 (1.69%)	1,646	24.21
6.	University of Sheffield, England	62 (1.54%)	898	14.48
7.	Saveetha Institute of Medical Technical Science, India	61 (1.51%)	244	4.0
8.	Egyptian Knowledge Bank, Egypt	58 (1.44%)	520	8.97
9.	Universidade Federal de Minas Gerais, Brazil	58 (1.44%)	994	17.09
10.	Nationwide Children’s Hospital, United States	55 (1.36%)	850	15.45

Authorship Patterns

**Figure 2:**
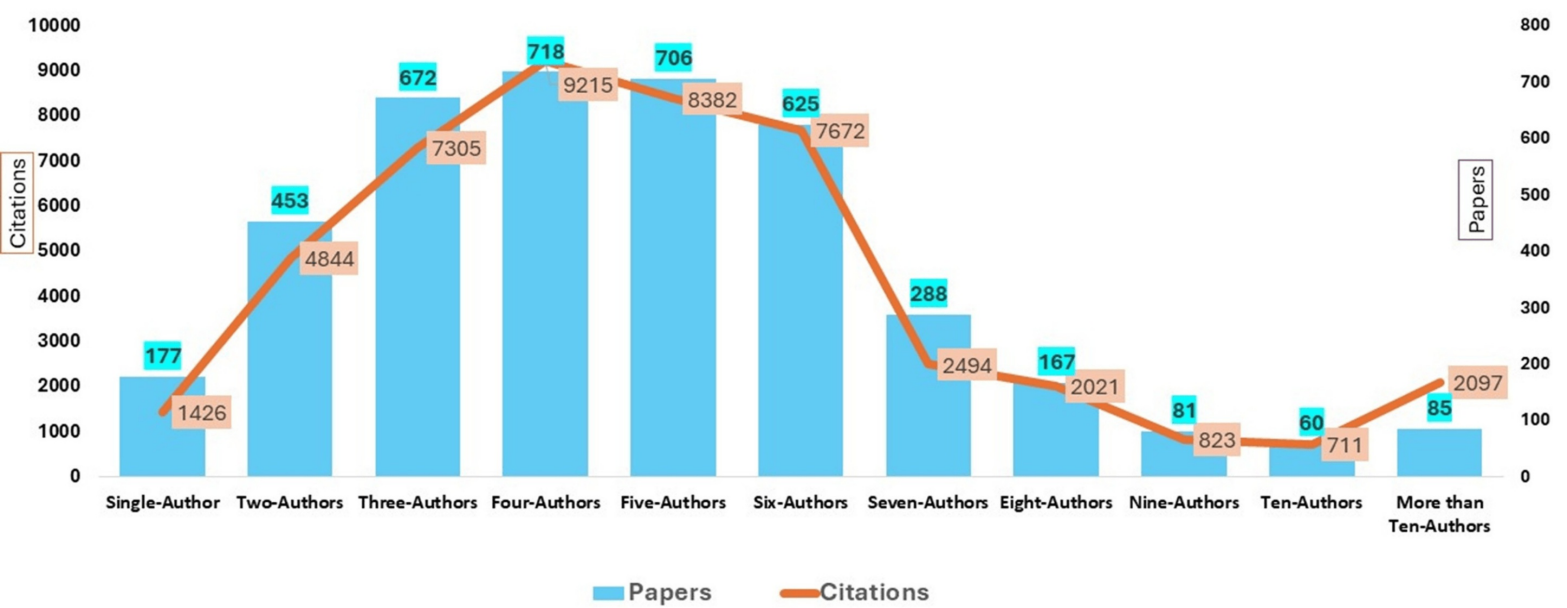
Authorship patterns with paper and citations The blue bars with blue-shaded boxes indicate the authorship pattern with the total number of papers. The orange lines with the orange-shaded boxes indicate total citations gained by a particular authorship pattern.

The examination of authorship patterns reveals a clear association between team size and research impact, as illustrated in Figure 2. Single-author papers are the least common (n = 177; 4.38%) and exhibited the lowest average citation of 8.06 per paper, suggesting limited visibility. As the number of authors increases, both productivity and citation performance tend to improve, with the highest volume of papers produced by teams of three to six authors (n = 2721; 67.48%). Four-author and six-author groups show strong average citation of 12.83 and 12.28 per paper, respectively, indicating that mid-sized collaborations are particularly influential. Although papers with more than 10 authors are relatively few, they demonstrate the highest average citation of 24.67 per paper, reflecting the significant visibility of large-scale, multicenter collaborative studies. Overall, these findings indicate that collaborative research generally results in more impactful scholarship compared to smaller or single-author outputs.

Authorship Ratio by Five-Year Intervals

The dataset demonstrates a clear upward trend in both research productivity and collaborative intensity over time. During the first interval (2005-2009), only 338 papers (8.38%) were published, authored by an average of 2.67 authors per paper. In the next interval (2010-2014), 16% of the papers (n = 641) were published, with an average of 4.17 authors per paper. In the third interval (2015-2019), 25% of the papers (n = 1007) were produced by an average of 4.65 authors per paper. Half of the papers (n = 2046; 50.74%) were published during the last interval (2020-2024) and had the highest average of authors at 5.13. Overall, a total of 19,126 authors, including multiple counts, contributed to 4,032 papers, with an average of 4.74 authors per paper.

Publications increased steadily from 8.38% (n = 338) in the first interval to 50.74% (n = 2046) in the last interval, reflecting a substantial expansion of research output in the field. This growth is accompanied by an even sharper rise in the number of contributing authors; the average number of authors per paper increased consistently from 2.67 to 5.13. This pattern indicates a shift toward larger and more collaborative research teams, suggesting that modern studies increasingly require multidisciplinary expertise, shared resources, and broader research networks. Overall, the analysis highlights both the rapid growth of scholarly production and the strengthening of a collaborative research culture over the past two decades.

Top Authors

A total of 13,527 distinct authors were identified, with 81.61% (n = 11,040) contributing to a single paper. Only 69 authors contributed to 10 or more papers. The top 10 most prolific authors are listed in Table 6. This table describes these productive authors and reveals notable differences in both research output and scholarly impact. Paul S. Casamassimo leads with 44 papers and a strong citation impact of 25.89, indicating both high productivity and significant influence. Similarly, Christian Splieth and Saul Martins Paiva demonstrate high average citation of 24.45 and 24.43 per paper, respectively, alongside substantial publication counts, signifying steady contributions. David John Manton from the University of Melbourne, Australia, stands out, despite having fewer papers than the top author (27 papers), claiming the highest average citation impact of 54.93 per paper, suggesting that the work is exceptionally influential and widely cited. In contrast, authors such as Kazuhiko Nakano, Rena Okawa, and John H. Langdon, while productive, exhibit comparatively low average citations, ranging from 2.46 to 4.08 per paper. This analysis reflects a diverse group of leading contributors, with some excelling in both volume and impact, while others contribute significantly to output but have lower citation visibility.

**Table 6:** Top 10 most productive authors

Serial No.	Name of Author	Affiliation	Total Papers	Total Citations	Citation Impact
1.	Casamassimo, Paul S.	Ohio State University, United States	44 (1.09%)	1,139	25.89
2.	Splieth, Christian	University Medicine of Greifswald, Germany	31 (0.76%)	758	24.45
3.	Nakano, Kazuhiko	Osaka University, Japan	30 (0.74%)	112	3.73
4.	Raggio, Daniela Prócida	University of São Paulo, Brazil	29 (0.71%)	593	20.45
5.	Paiva, Saul Martins	Universidade Federal de Minas Gerais, Brazil	28 (0.69%)	684	24.43
6.	Manton, David John	University of Melbourne, Australia	27 (0.66%)	1,483	54.93
7.	Okawa, Rena	University of Osaka, Japan	26 (0.64%)	106	4.08
8.	Langdon, John H.	University of Indianapolis, United States	26 (0.64%)	64	2.46
9.	Scully, C.	University of London, England	25 (0.62%)	132	5.28
10.	Townsend, Janice A.	Ohio State University, United States	24 (0.59%)	124	5.17

Top 20 Keywords

**Figure 3:**
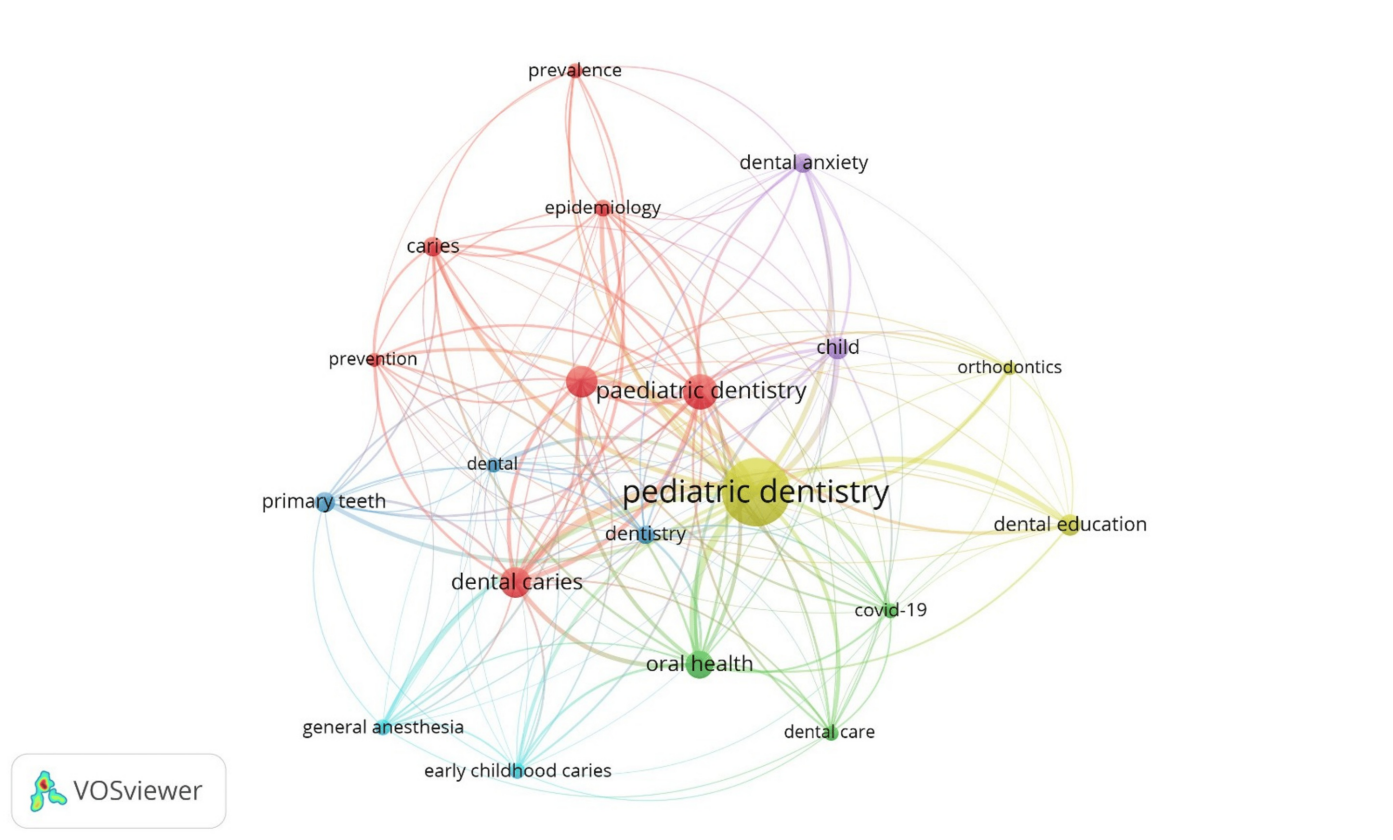
Co-occurence network of top 20 keywords

A total of 6,308 keywords have been utilized by the authors across 4,032 papers, with 73% (n = 4,596) of these keywords appearing only once in the dataset. The co-occurrence network of the top 20 keywords, as shown in Table 7 and Figure 3, reveals that research in PD is primarily focused on core thematic areas such as pediatric dentistry/paediatric dentistry, children, and oral health. These themes exhibit the highest occurrences and strongest link strengths, indicating their dominant role in the literature network. Caries-related terms - including dental caries, caries, and early childhood caries - constitute a major cluster, underscoring that the epidemiology, prevention, and management of dental caries remain central research priorities in this field. Population-specific keywords such as "Children," "Child," and "Primary Teeth" further highlight the demographic relevance of the discipline. Clinical management themes, including dental anxiety, general anesthesia, and dental care, demonstrate significant connectivity, reflecting ongoing research interest in behavioral management and the complexities of treating young patients. Public health and educational aspects, represented by Oral Health and Dental Education, also show strong interconnections, suggesting an increasing focus on preventive strategies and the training of dental professionals. Furthermore, the mention of COVID-19 indicates the impact of recent global health challenges on pediatric dental research. Overall, the distribution and link strength of the keywords illustrate a well-connected research landscape dominated by themes of caries, child-centered care, clinical management, and public health within PD.

**Table 7:** Top 20 keywords

Serial No.	Keywords	Occurrences	Total Link Strength
1.	Pediatric dentistry	1,351	819
2.	Paediatric dentistry	368	249
3.	Children	296	244
4.	Dental caries	277	330
5.	Oral health	230	325
6.	Child	145	159
7.	Dental education	133	99
8.	Primary teeth	125	93
9.	Caries	112	125
10.	Dental anxiety	108	116
11.	Dentistry	107	114
12.	Epidemiology	86	127
13.	Early childhood caries	77	79
14.	General anesthesia	73	91
15.	COVID-19	69	89
16.	Prevalence	69	44
17.	Dental care	66	88
18.	Dental	63	63
19.	Prevention	62	91
20.	Orthodontics	60	61

In bibliometric analysis, keyword co-occurrence reveals how themes are related. When certain keywords repeatedly appear together in a dataset, it indicates that these topics are closely linked, often explored together, or belong to the same thematic area. Total link strength represents the overall strength of connections that a keyword has with other keywords in a co-occurrence network.

Languages and Funding Sources

English was the primary language used in PD research, comprising 96.47% (n = 3891) of all publications. Following English, Spanish and Portuguese contribute 53 and 45 papers, respectively. The remaining 141 papers (3.53%) published in eight different languages - German (8), Polish (8), French (7), Italian (7), Turkish (7), Croatian (3), Serbian (3), and Indonesian (1) -make relatively minor contributions, with publication counts ranging from eight to just one.

Among the leading five funding organizations, the United States Department of Health and Human Services ranks first, having supported 115 papers. It is followed by the Coordenacao de Aperfeiçoamento de Pessoal de Nível Superior, which has funded 109 papers, and the National Institutes of Health, with 98 papers. The fourth and fifth spots are held by the Conselho Nacional de Desenvolvimento Científico e Tecnológico and Japan’s Ministry of Education, Culture, Sports, Science and Technology, which have funded 80 and 76 publications, respectively.

Discussion

All branches of biomedical sciences, including dentistry, are continuously evolving, resulting in changes in how information is generated, accessed, and interpreted. As a consequence, practitioners and researchers must remain informed about emerging developments. The increasing volume of scientific literature has enhanced the dissemination of research findings [21,22]. Bibliometric analyses are commonly employed to assess the scholarly impact of publications. These analyses enable the evaluation of a specific topic's global or national standing, the identification of research trends, and the highlighting of areas that need further investigation. Such insights offer valuable guidance for future studies [23,24]. The dataset for the present study was sourced from the WoS, a comprehensive database that provides bibliographic and citation evidence for high-quality publications across various academic fields, including dentistry. WoS is a crucial resource for bibliometric studies due to its coverage of influential journals, books, and conference proceedings [8].

PD is a specialized branch of dentistry that addresses the oral health needs of infants, children, and adolescents through preventive and therapeutic care. However, its significance is not reflected in the amount of research attention it receives. A study evaluated 2,574 papers published between 2009 and 2023 in five Saudi dental journals and found that only 49 (5.74%) focused on PD, indicating a lack of research in this field [20]. This limited quantity may stem from the fact that many specialized PD articles are published in discipline-specific international journals rather than in general dental journals.

The current study evaluated 4,032 papers on PD published between 2005 and 2024. The findings reveal a consistent upward trend in publication productivity, increasing from 35 papers in 2005 to 508 in 2024, which corresponds to an average growth rate of 16.87%. Approximately one-fourth of the publications were released in the first decade, while the majority appeared after 2014, indicating a growing interest, improved research infrastructure, and increased scholarly activity in the field. Citation analysis indicates that earlier studies (2009-2014) received higher citation counts, likely due to their longer exposure and the attention given to foundational topics. Although recent years show lower citation metrics, this phenomenon is primarily attributed to the recency effect rather than a decline in research quality [25]. A separate study assessed 2,848 publications from the *Journal of Dentistry* and the *Pediatric Dentistry* journal published between 1969 and 1988. In the first decade (1969-1978), 15.90% of papers were published; in the second decade (1979-1988), 39.10% were published; and in the third decade (1989-1998), 45% of the papers were published [12].

Analysis of bibliometric indicators (Table 2) demonstrates that 43.5% (n = 1054) of PD papers were open access, while 56.5% (n = 2278) were subscription-based, with subscription papers slightly outperforming in citation impact. Most publications consisted of research articles (88.5%; n = 3569), whereas review articles (11.5%; n = 463) exhibited a significantly higher citation impact (20.27 vs. 10.59), reflecting their broader relevance. Review articles typically attract higher citation counts than original research due to their ability to consolidate and summarize extensive evidence, making them widely applicable and accessible to a larger readership [26]. Nainar analyzed papers published in two dental journals over a 30-year period, finding that 71% were related to descriptive studies or case reports, while only 6% were randomized controlled trials [12]. Another study indicated that most papers published in the *Brazilian Journal of Pediatric Dentistry* from 1998 to 2007 focused on case reports (33%), cross-sectional studies (30%), and literature reviews (23%), with only 2.4% addressing randomized controlled trials [10]. In our study, 73% (n = 2939) of the papers appeared in dental journals, which also had a higher citation impact (12.43 vs. 9.56), suggesting that discipline-specific journals tend to attract more attention and citations. A bibliometric study on pulpotomy further confirmed this trend, showing that 70% of the papers were published in dental journals, which achieved a higher citation ratio [9].

Journals play a crucial role in scholarly communication by facilitating the dissemination of research, ensuring the credibility of knowledge through peer review, and promoting academic discussion among researchers [27]. Since the inaugural issue of the pioneering *American Journal of Dental Science* in 1839, the number of dental journals - encompassing both general and specialized fields - has steadily increased [28]. Our findings indicate that 4,032 papers were published across 552 sources, with over half (54%) of these sources contributing only one paper each. Notably, the top 10 sources accounted for 44% of the total output. The *Pediatric Dental Journal *(n = 311) and *Pediatric Dentistry* (n = 277) exhibited the highest productivity, while journals such as the *International Journal of Paediatric Dentistry* (22.21) and *European Archives of Paediatric Dentistry* (20.32) demonstrated significant average citation per paper, despite having fewer publications, indicating their strong scholarly influence. García et al. reviewed 3,027 papers published in four PD journals from 2008 to 2020, finding that the majority were in the *International Journal of Paediatric Dentistry* (27.5%), followed by the *Journal of Clinical Pediatric Dentistry* (26.2%) [15]. Another study on the 100 most cited articles in PD reported that most were published in* Paediatric Dentistry* (n = 45) and the *International Journal of Paediatric Dentistry* (n = 27) [14]. Additionally, a study focusing on PD research from Turkiye indicated that the *Journal of Clinical Pediatric Dentistry* had the highest number of publications (n = 159), followed by *Dental Traumatology* (n = 82) and the *Journal of Dentistry for Children* (n = 78) [19]. Ohta et al. [11] examined the publication records of the *Pediatric Dentistry* journal, while Poletto et al. [10] analyzed papers published in the *Brazilian Journal of Pediatric Dentistry*​​​​​.

Developed countries typically produce more publications due to well-established research institutions, stronger research infrastructure, and longstanding traditions of scholarly activity [29]. Our study found that authors from 103 countries contributed to the 4,032 PD papers, with 58 countries producing at least 10 publications. The United States was the most productive (18.75%; n = 756), followed by Brazil (12.25%; n = 494) and India (8.65%; n = 349). Despite contributing a smaller share (2.85%), Australia achieved the highest citation impact (31.55 citations per paper), with England and Germany also demonstrating a strong impact.

García et al. analyzed the 3,027 papers published in four PD journals and found that the United States produced the most articles, followed by Brazil, Italy, and India, based on the affiliation of the first author [15]. Another study on pediatric dental sedation indicated that 35% of the literature was published by authors from the United States, followed by England (13.6%) [16]. The United States also ranked at the top regarding the 100 most cited articles in PD, contributing 43% of the papers. However, Sweden, with six articles, achieved the highest citation impact (123.33), followed by Jordan (113.50) [14]. Mahyaddinova et al. examined 342 papers on neurodevelopmental disorders in PD, finding that one-third of the literature originated from the United States, with India (12%), China (7%), and England (5%) following [17].

The majority of research is produced by universities, which serve as key centers of investigation due to their robust academic resources and vibrant scientific communities [30]. The Baltimore College of Dental Surgery, established in 1840 as the world’s first dental school solely dedicated to awarding dentistry degrees, merged with the University of Maryland in 1923. Since that time, the number of dental institutions has steadily increased [31]. Our study findings indicate that 3,200 research organizations contributed to 4,032 papers; most (66.28%; n = 2121) produced only a single publication, while 156 institutions generated 10 or more papers. The Universidade de São Paulo was the most productive institution (n = 118; 2.93%), followed by the University System of Ohio (n = 98) and the University of London (n = 96; 2.38%). In terms of research impact, the University of Washington ranked highest with an average of 26.04 citations per paper, followed by the University of North Carolina (24.21) and the University of London (18.7). Zhang et al. reviewed papers on pediatric dental sedation and found that the Federal University of Goiás produced the highest number of papers (n = 42), followed by the University of Washington (n = 20). However, the University of Washington had the top citation impact, followed by Ohio State University [16]. A recent study focusing on PD research in Turkiye indicated that Hacettepe University contributed most of the research, with international research collaboration found in only 5% of cases, mostly with universities from the United States, Brazil, and England [19]. Poletto et al. examined papers published in the *Brazilian Journal of Pediatric Dentistry* and found that most papers were contributed by authors from São Paulo (40%), followed by those from Rio de Janeiro (17%) [10].

Analysis of authorship patterns indicates that collaborative research, particularly those involving mid to large-sized teams, tends to increase both research output and academic influence compared to single-author works [32]. Our findings on PD research reveal that collaborative research yields a higher impact, with mid-sized teams (three to six authors) producing the majority of papers and large teams (more than 10 authors) achieving the highest citation rates, while single-author studies exhibit the lowest impact. A study reviewed PD research from Turkiye and found that only 47 papers (2.41%) were authored by a single researcher [19]. García et al. calculated the number of papers published in four PD journals and reported that the average number of authors per article was 4.4 ± 1.9. An evident trend of increasing authorship was observed over the past decade: the average in 2020 was 4.8 authors per article, compared to an average of 3.7 authors per article 10 years prior [15].

Over time, both research productivity and collaborative efforts in PD have increased [33]. The results of our study indicated a consistent rise in productivity and collaboration in PD research, measured in five-year intervals. The percentage of published papers grew from 8.38% (n = 338) during the period of 2005-2009 to 50.74% (n = 2046) in the years 2020-2024. Additionally, the average number of authors per paper increased from 2.67 to 5.13, indicating a shift toward larger, multidisciplinary teams. Overall, an average of 4.74 authors per paper has been recorded, emphasizing the rapid growth of scholarly output and the development of an increasingly collaborative research culture over the past two decades.

Skilled and accomplished researchers are crucial for the advancement of science, as they provide rigor, foster innovation, and produce influential work that enhances the credibility and development of their disciplines [34]. Our study identified a total of 13,527 authors contributing to PD research, with 81.6% (n = 11,040) of these authors publishing only a single paper, while 69 authors produced 10 or more papers. Among the top contributors, Paul S. Casamassimo led in productivity with 44 papers and a citation impact of 25.89. In contrast, David John Manton achieved the highest citation impact of 54.93 with fewer papers (n = 27), reflecting his significant influence. Other prolific authors, such as Christian Splieth and Saul Martins Paiva, combined substantial output with high impact. A related study on pulpotomy revealed that 738 papers were produced by 3,017 authors, with 88% (n = 2,656) contributing to only a single paper, while a small group of authors (n = 361; 12%) contributed to more than one paper [9].

Keyword co-occurrence is a critical bibliometric tool for identifying core themes, uncovering emerging trends, and mapping knowledge clusters within a research field [35]. Our study revealed that a total of 6,308 keywords were used across 4,032 PD papers, with 73% (n = 4596) appearing only once. The co-occurrence analysis of the top 20 keywords highlights central themes such as PD, children, and oral health. Caries-related terms are prevalent, underscoring the importance of epidemiology, prevention, and management, while clinical management keywords reflect research on behavioral aspects and treatment strategies. Population-specific and public health terminology indicates a focus on child-centered care and preventive measures. Overall, the keyword analysis illustrates a well-connected research landscape centered on caries, clinical management, child care, and public health within the field of PD.

Yang et al. examined records of PD indexed in PubMed and found that most papers focused on oral medicine, followed by restorative dentistry, while the fewest papers addressed endodontics [13]. A study published in the *Brazilian Journal of Pediatric Dentistry* reported that cariology and restorative dentistry were the top areas of interest [10]. Research in the *Pediatric Dentistry* journal revealed that cariology (12.7%), restorative dentistry (10.6%), and systemic diseases (9.4%) were leading research areas [11]. A study from Turkiye indicated that common keywords included “children” (n = 178), “dental care” (n = 135), and “pediatric dentistry” (n = 98) [19]. The analysis of the 100 most cited articles in PD revealed that caries/early childhood caries, pulp therapy, and dental trauma were among the top subjects [14]. Additionally, Zhang et al. found that in their study of pediatric dental sedation, the most common topics in the keyword co-occurrence analysis were dental anxiety, conscious sedation, and dental caries [16].

This review employs the WoS as its primary citation database. Historically, WoS has established the criteria for counting citations while emphasizing selective content coverage. This selective approach aligns with Bradford’s Law (1934), which identifies influential journals. WoS values the quality of coverage over sheer quantity and facilitates bibliometric analyses. It continues to be the leading database for citation analysis across various disciplines [36].

Limitations

This study has several limitations. First, it relied on specific keywords to identify PD literature, potentially missing relevant studies that did not include those terms. Only articles and review papers were included, while other document types were excluded, which may have constrained the scope of the analysis. Furthermore, this study did not differentiate between clinical and non-clinical content or assess levels of evidence; it primarily focused on quantitative bibliometric data.

This bibliometric investigation solely employed the WoS database, perhaps excluding relevant articles not indexed by WoS. Future research could benefit from broader search strategies, the inclusion of additional databases such as PubMed and Scopus, the combination of gray literature, and qualitative assessments to explore thematic distribution and research trends in PD.

## Conclusions

This bibliometric analysis indicates a significant increase in PD publications from 2005 to 2024, driven by growing academic interest, enhanced research capacity, and improved collaboration. The United States, Brazil, and India emerged as the leading contributors, while countries like Australia and England demonstrated strong citation performance. The most impactful studies were published in specialized dental journals and produced by mid- to large-sized research teams. Keyword patterns reveal a continued emphasis on caries, preventive dentistry, and clinical management as primary research areas. Despite overall growth, research contributions remain uneven across countries and institutions, with a majority of authors contributing only a single paper. Future investigations should utilize broader databases, expanded search methodologies, and qualitative assessments to gain deeper insights into global research patterns in PD.
